# On K-Banhatti indices and entropy measure for rhodium (III) chloride via linear regression models

**DOI:** 10.1016/j.heliyon.2023.e20935

**Published:** 2023-10-16

**Authors:** Mazhar Hussain, Muhammad Kamran Siddiqui, Muhammad Farhan Hanif, Hasan Mahmood, Zohaib Saddique, Samuel Asefa Fufa

**Affiliations:** a Department of Mathematics, COMSATS University Islamabad, Lahore Campus, Pakistan; b Abdus Salam School of Mathematical Sciences, Government College University, Lahore, Pakistan; c Department of Mathematics, Government College University, Lahore, Pakistan; d Department of Chemistry, University of the Punjab, Quaid-i-Azam Campus Lahore, Pakistan; e Department of Mathematics, Addis Ababa University, Addis Ababa, Ethiopia

**Keywords:** Degree-based Banhatti indices, Rhodium chlorid, Degree of vertex, Shannon entropy

## Abstract

Rhodium (III) chloride is a metallic compound characterized by its shiny and silvery-white appearance. It possesses high reflectivity and exhibits excellent resistance to corrosion. This makes it a popular choice for applications such as plating materials in jewelry and other decorative items, imparting a lustrous and reflective surface to the coated objects. Topological indices are numerical parameters employed to characterize the topology of a molecular structure. These indices are derived from the connectivity of atoms within the molecule and serve as predictors for various molecular properties, including reactivity, stability, and solubility. On the other hand, the Shannon entropy of a graph finds extensive applications in network science. It is utilized in the analysis of diverse networks, such as social networks, biological networks, and transportation networks. The Shannon entropy allows for the characterization of a network's topology and structure, aiding in the identification of crucial nodes or structures that play significant roles in network functionality and stability. In this paper, our primary objective is to compute different K-Banhatti indices and employ them to evaluate the entropy measure of Rhodium (III) chloride $RhCl_{3}$. Additionally, we conducted an examination through linear regression analysis involving various indices and entropies associated with Rhodium chloride. Moreover, we established a correlation between degree-based Banhatti indices and entropies via the line fit method.

## 1 Introduction

A graph consists of a collection of vertices (also referred to as nodes or points) and a collection of edges (also known as links or arcs) that establish connections between pairs of vertices. This branch (Graph Theory) of mathematics provides a framework for analyzing and comprehending intricate networks and relationships across various disciplines, such as computer science, engineering, physics, and social sciences. It enables the exploration of concepts like connectivity, paths, cycles, optimization, and many other properties associated with graphs and their applications.

In chemistry, graph theory finds extensive utility in representing the molecular structure of chemical compounds as graphs. In a chemical graph, the vertices correspond to the atoms within the molecule, while the edges signify the bonds linking these atoms. By scrutinizing the topology and properties of chemical graphs, chemists can acquire valuable insights into the physical and chemical characteristics of molecules, including reactivity, stability, and biological activity. Chemical graph theory boasts numerous applications in chemistry and related disciplines, such as drug design, chemical synthesis, materials science, and catalysis. For instance, it can aid in the prediction of properties for newly synthesized compounds, the development of more efficient catalysts for chemical reactions, and the comprehension of structure-activity relationships pertaining to drugs and bioactive molecules.

Throughout the paper, the graph is denoted as *G*. If e=μυ represents an edge connecting vertices *μ* and *υ*, the degree of the edge *e* in *G*, denoted as $\theta_{e}$, is defined as $\theta_{e}=d(\mu )+d(\upsilon )-2$. Kulli [18] examined the K-Banhatti index of graphs. Kulli [19] also investigated the multiplicative and hyper K-Banhatti indices of nanotubes. Wang et al. [38] discussed the K-Banhatti indices of acid curcumin. Rehman [31] examined the K-Banhatti indices of nanotubes. Anjum et al. [2] discussed the K-Banhatti and Hyper K-Banhatti indices of nanotubes. Table 1 presents various Banhatti Indices.

**Table 1:** Different K-Banhatti Indices.

K-Banhatti Indices	Notation	Mathematical Formula
The First K-Banhatti Index [20]	*B*_1_(*G*)	∑_*μe*_[*d*(*μ*)+*θ*_(*e*)_]
The second K-Banhatti Index [20]	*B*_2_(*G*)	∑_*μe*_[*d*(*μ*)×*θ*_(*e*)_]
The first K hyper-Banhatti Index [21]	*HB*_1_(*G*)	$\sum_{\mu e}{\left[d(\mu )+\theta_{\left(e\right)}\right]}^{2}$
The second K hyper-Banhatti Index [21]	*HB*_2_(*G*)	$\sum_{\mu e}{\left[d(\mu )\times \theta_{\left(e\right)}\right]}^{2}$
The K-Banhatti harmonic Index [22]	*H*_*b*_(*G*)	$\sum_{\mu e}[\frac{2}{d(\mu )+\theta_{\left(e\right)}}]$
The first hyper Revan Index [21]	*ηR*_1_(*G*)	∑_*e*=*μυ*_[*τ*(*μ*)+*τ*(*υ*)]^2^
The second hyper Revan Index [21]	*ηR*_2_(*G*)	∑_*e*=*μυ*_[*τ*(*μ*)×*τ*(*υ*)]^2^
The third Revan Index [23]	*ηR*_3_(*G*)	∑_*e*=*μυ*_[*τ*(*μ*)−*τ*(*υ*)]
The first Revan vertex Index [6]	*R*_1_(*G*)	$\sum_{e=\mu \upsilon }[\tau {\left(\bar{\mu }\right)}^{2}]$

Where τ(μ) obtain by adding degree of *μ* and degree *υ* and then subtract degree *μ*, similarly we obtain τ(υ) by adding degree of *μ* and degree *υ* and then subtract degree *υ*, $\tau (\bar{\mu })=\Delta (G)+\delta (G)-d(\mu )$, where Δ(G) be the maximum degree, δ(G) be the minimum degree and d(μ) be the degree of vertex *μ*.

**Figure 1:**
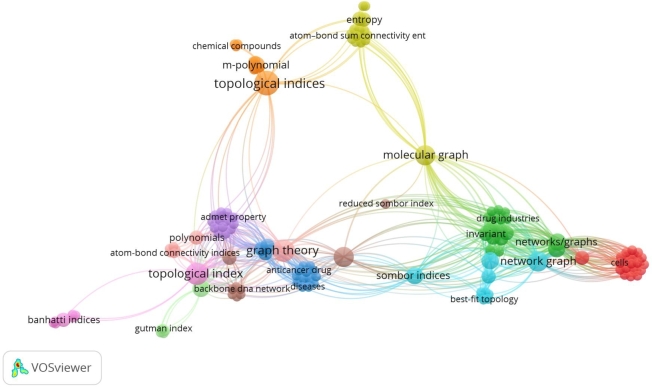
Bibliometric analysis of occurrence keyword (Banhatti Index) in publications.

The Banhatti Index keywords in different areas, such as graph entropy, complex networks, transfer entropy, inclusion degree, entropy weight, and agent-based modeling and risk evaluation, respectively, were used. We presented the bibliometric analysis of Banhatti Index keywords in Fig. 1 as shown below. This Fig. 1 illustrates that the concept of the Banhatti Index is related to many research areas in recent years and interlinks with many other topological indices.

**Figure 2:**
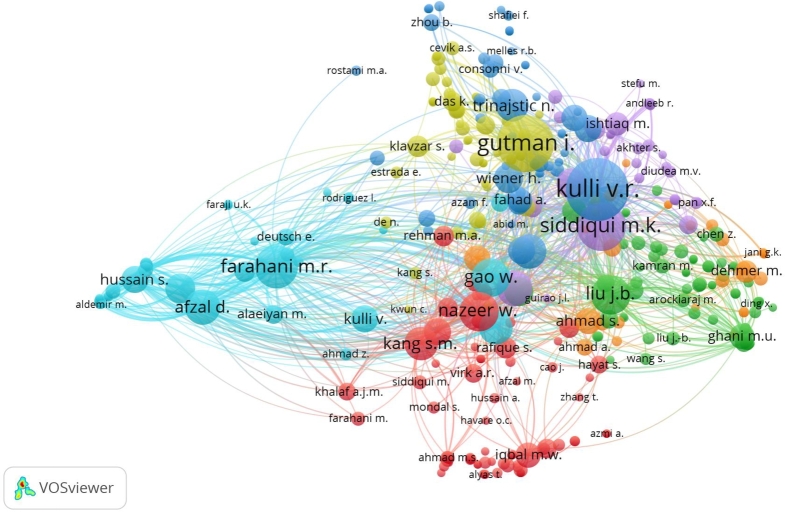
Bibliometric analysis of the citations of authors-wise research based on K-Banhatti Index.

We have presented a bibliometric analysis of different authors who work on the topic of the Banhatti Index. We use the Scopus database for this analysis with 845 research articles. This figure shows that different coauthors are working on this topic and writing articles together. Moreover, the main idea of the Banhatti Index is introduced by Kulli in 2016. Fig. 2 shows that most of the work is done by Kulli and its co-authors.

Shannon entropy, named after the mathematician Claude Shannon, measures a system's uncertainty or randomness. It is commonly used in information theory, communication theory, and other fields, such as physics, chemistry, and biology. Shannon first introduced entropy in his renowned article [34], which said that the entropy of a probability distribution is viewed as a measure of the ambiguity of a system. It was created for analyzing graph structure data and the related chemical networks. The aforementioned graph entropy measures come in a wide variety of specific forms [29]. Zhang et al. [39], [40] discuss the topological indices of generalized bridge molecular graphs, Carbon Nanotubes, and products of chemical graphs. Shazia et al. [26] computed the Entropy Measures for Isomeric Natural Polymers.

Zhang et al. [41], [42] provided the physical analysis of heat for the formation and entropy of Ceria Oxide. Dehmer [11] introduced information-based function graph entropies that examine properties and capture structural information. Zhao et al. [37] reported the research of graphitic carbon nitride based on molecular topological indices. Arockiaraj, M., et al. [4], [5] discussed the entropies for different structures. Zhang et al. [43], [44] gives an analysis of different Molecular Structures using Topological indices. Kavitha, S. R. J., et al. [17] discussed the topological characterization and graph entropies of tessellations of kekulene structures.

Shannon groundbreaking work in the late 1940*s* marked the inception of modern information theory [34]. Information theory has been widely used in biology and chemistry after seeing early linguistics and electrical engineering applications. Entropy measurements play a crucial role in these applications [28]. The structural complexity of graphs has been measured using Rashevsky's notion of graph entropy [12].

In their 2014 publication, Chen et al. [9] introduced the notion of entropy for edge-weighted graphs. They proposed a simple graph denoted as G(V(G);E(G);ψ(μν)), where ψ(μν) represents the edge weight of the edge ψ(μν) in *G*. Eq. (1) defines the entropy of the edge-weighted graph.

(1) $$E_{\psi }(G)=-\sum_{\mu \nu \in E(G)}\frac{\psi (\mu \nu )}{\sum_{\mu \nu \in E(G)}\psi (\mu \nu )}\log[\frac{\psi (\mu \nu )}{\sum_{\mu \nu \in E(G)}\psi (\mu \nu )}]$$

## 2 Rhodium (III) chloride structure

**Figure 3:**
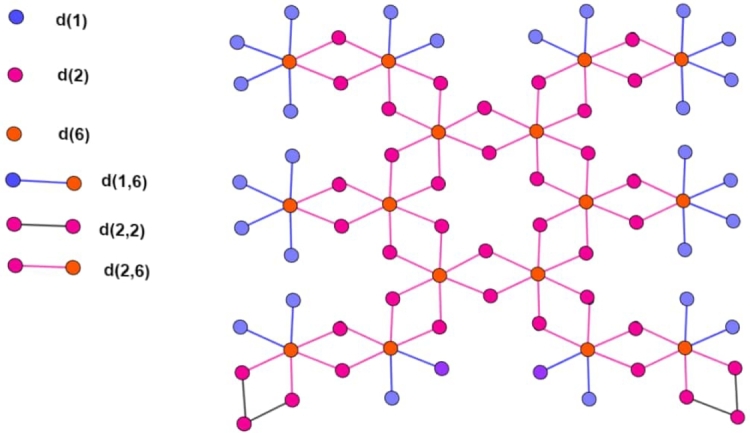
Unit Structure.

Due to the distinctive feature of a variable oxidation state, transition metal forms a variety of salt with other periodic table elements, such as halogens. Rhodium chloride is one of them, where transition metal rhodium (Rh) forms a salt with chlorine from the halogen group. In this inorganic salt oxidation state of rhodium is (III). This inorganic salt is diamagnetic, and it has octahedral geometry with three chloride ions bonded with Rh (III) metal centers, as shown in Fig. 3. It is present in both hydrated and anhydrous forms, and its color depends upon the number of water molecules present with it. In its anhydrate form, it's in brown solid and octahedral molecular geometry [10].

Rhodium chloride with three molecules of water is soluble in commonly used solvents, and it's widely used in the laboratory for preparative purposes. Anhydrous Rhodium chloride is not soluble, so it is not used in laboratories [14]. The sodium salt of rhodium chloride is used to synthesize rhodium chloride through ion exchange chromatography. The trisodium salt of rhodium is obtained from common ores of platinum and iridium group metals. Hydrated rhodium is collected after crystallization of product [8]. To prepare anhydrous rhodium chloride, both are reacted in metallic form at the higher temperature of $200-300^{\circ }$ [32]. Rhodium chloride trihydrate has been used to synthesize various complexes, despite its complexity in the formation of its solutions. It reacts with various chemical species to produce a variety of complexes such as ammonia, pyridine, phosphines, thioethers, arsines, acetylacetone, 1,5-cyclooctadiene, and alkenes [13], [15], [24], [27], [30], [33], [35], [36]. Being a transition metal salt and having a variable oxidation state leads to the application of rhodium chloride in various catalytic applications such as asymmetric ring opening reactions [25], activation of C-H bond in organic compounds [16], decomposition of nitrous oxides [7], conversion of chlorobenzene [1]. The considered structure to be the subgraph of the structure [3].

**Figure 4:**
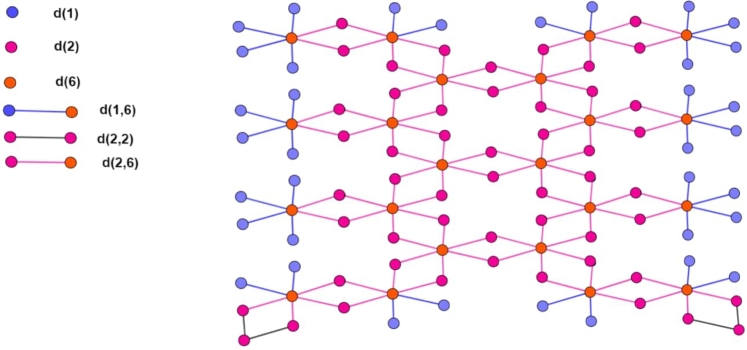
Two Copies of Structure.

**Figure 5:**
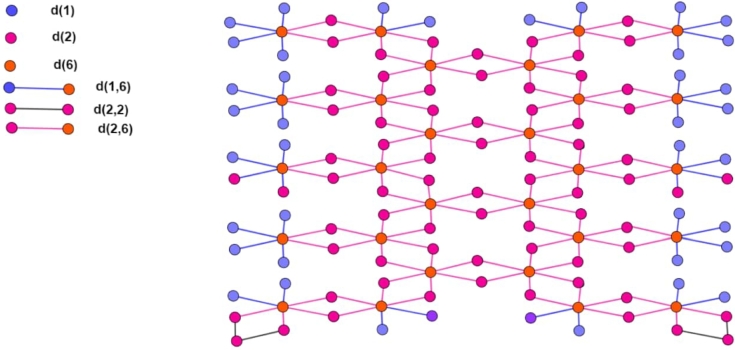
Three Copies of Structure.

Let *n* be the number of the unit cells, which are arranged linearly. In Figure 3, Figure 4, Figure 5, the number of vertices of degree 1 is represented by blue color denoted by d(1), the number of vertices of degree 2 is represented by red color denoted by d(2), and the number of vertices of degree 6 is represented by purple color denoted by d(6). The cardinality of vertices of degree 1, 2 and 6 is 8n+20, 14n+24 and 6n+10. The cardinality of edges between vertices of degrees (1,6) is 8n+20 and is denoted by d(1,6). The cardinality of edges between vertices of degrees (2,2) is 4 and is denoted by d(2,2). The cardinality of edges between vertices of degrees (2,6) is 14n+22 and is denoted by d(2,2). The order and size of the Rhodium (III) Chloride molecular graph are 28n+54 and 22n+46, respectively. The vertex and edge partition are dedicated in Table 2 and Table 3 respectively.

**Table 2:** Division of vertices.

*d*(*μ*)	*Frequency*	$\tau_{\left(\bar{\mu }\right)}$
1	8*n* + 20	6
2	14*n* + 24	5
6	6*n* + 10	1

**Table 3:** Division of edges.

(*d*(*μ*),*d*(*υ*))	*Frequency*	*θ*_(*e*)_	*τ*_(*μ*)_	*τ*_(*υ*)_
(1,6)	8*n* + 20	5	6	1
(2,2)	4	2	5	5
(2,6)	14*n* + 22	6	5	1

## 3 Methodology

We computed K-Banhatti indices using various techniques, including edge partitioning, vertex degree approaches, neighborhood degree counts, and combinatorial computation. After that, we compute the entropy using the K-Banhatti indices. We make use of Matlab to generalize and validate calculations. To perform correlation analysis, specialized software called SPSS was used.

## 4 Main results

We performed calculations for several K-Banhatti Indices in this section and evaluated our numerical results. We also visually illustrated the response of each topological index to different parameter values. To achieve the desired outcomes, we utilized various strategies. Using values Table 3 in Table 1, we obtain different K-Banhatti indices as follows.

• The first K-Banhatti index

$$B_{1}(G)=\sum_{\mu e}[d(\mu )+\theta_{\left(e\right)}]B_{1}(G)=(8n+20)[(1+5)+(6+5)]+(4)[(2+2)+(2+2)]+(14n+22)[(2+6)+(6+6)]=(8n+20)[(6)+(11)]+(4)[(4)+(4)]+(14n+22)[(8)+(12)]=(8n+20)(17)+(4)(8)+(14n+22)(20)=416n+812.$$

• The second K-Banhatti index

$$B_{2}(G)=\sum_{\mu e}[d(\mu )\times \theta_{\left(e\right)}]B_{2}(G)=(8n+20)[(1\times 5)+(6\times 5)]+(4)[(2\times 2)+(2\times 2)]+(14n+22)[(2\times 6)+(6\times 6)]=(8n+20)[(5)+(30)]+(4)[(4)+(4)]+(14n+22)[(12)+(36)]=(8n+20)(35)+(4)(8)+(14n+22)(48)=952n+1788.$$

• The first hyper K-Banhatti index

$$HB_{1}(G)=\sum_{\mu e}{\left[d(\mu )+\theta_{\left(e\right)}\right]}^{2}\;HB_{1}(G)=(8n+20)[{\left(1+5\right)}^{2}+{\left(6+5\right)}^{2}]+(4)[{\left(2+2\right)}^{2}+{\left(2+2\right)}^{2}]+(14n+22)[{\left(2+6\right)}^{2}+{\left(6+6\right)}^{2}]=(8n+20)[{\left(6\right)}^{2}+{\left(11\right)}^{2}]+(4)[{\left(4\right)}^{2}+{\left(4\right)}^{2}]+(14n+22)[{\left(8\right)}^{2}+{\left(12\right)}^{2}]=(8n+20)[(36)+(121)]+(4)[(16)+(16)]+(14n+22)[(64)+(144)]=(8n+20)(157)+(4)(32)+(14n+22)(208)=4168n+7844.$$

• The second hyper K-Banhatti index

$$HB_{2}(G)=\sum_{\mu e}{\left[d(\mu )\times \theta_{\left(e\right)}\right]}^{2}\;HB_{2}(G)=(8n+20)[{\left(1\times 5\right)}^{2}+{\left(6\times 5\right)}^{2}]+(4)[{\left(2\times 2\right)}^{2}+{\left(2\times 2\right)}^{2}]+(14n+22)[{\left(2\times 6\right)}^{2}+{\left(6\times 6\right)}^{2}]=(8n+20)[{\left(5\right)}^{2}+{\left(30\right)}^{2}]+(4)[{\left(4\right)}^{2}+{\left(4\right)}^{2}]+(14n+22)[{\left(12\right)}^{2}+{\left(36\right)}^{2}]=(8n+20)[(25)+(900)]+(4)[(16)+(16)]+(14n+22)[(144)+(1296)]=(8n+20)(925)+(4)(32)+(14n+22)(1440)=27560n+50308.$$

• The K-Banhatti harmonic index

$$H_{b}(G)=\sum_{\mu e}[\frac{2}{d(\mu )+\theta_{\left(e\right)}}]H_{b}(G)=(8n+20)[\frac{2}{\left(1+5\right)}+\frac{2}{\left(6+5\right)}]+(4)[\frac{2}{\left(2+2\right)}+\frac{2}{\left(2+2\right)}]+(14n+22)[\frac{2}{\left(2+6\right)}+\frac{2}{\left(6+6\right)}]=(8n+20)[\frac{2}{\left(6\right)}+\frac{2}{\left(11\right)}]+(4)[\frac{2}{\left(4\right)}+\frac{2}{\left(4\right)}]+(14n+22)[\frac{2}{\left(8\right)}+\frac{2}{\left(12\right)}]=(8n+20)[\frac{17}{33}]+(4)[\frac{4}{4}]+(14n+22)[\frac{5}{12}]=\frac{219}{22}n+\frac{1549}{66}.$$

• The first Revan index

$$\eta R_{1}(G)=\sum_{e=\mu \upsilon }{\left[\tau (\mu )+\tau (\upsilon )\right]}^{2}\;\eta R_{1}(G)=(8n+20)[{\left(6+1\right)}^{2}]+(4)[{\left(5+5\right)}^{2}]+(14n+22)[{\left(5+1\right)}^{2}]=(8n+20)[{\left(7\right)}^{2}]+(4)[{\left(10\right)}^{2}]+(14n+22)[{\left(6\right)}^{2}]=(8n+20)[(49)]+(4)[(100)]+(14n+22)[(36)].=896n+2172.$$

• The second Revan index

$$\eta R_{2}(G)=\sum_{e=\mu \upsilon }{\left[\tau (\mu )\times \tau (\upsilon )\right]}^{2}\;\eta R_{2}(G)=(8n+20)[{\left(6\times 1\right)}^{2}]+(4)[{\left(5\times 5\right)}^{2}]+(14n+22)[{\left(5\times 1\right)}^{2}]=(8n+20)[{\left(6\right)}^{2}]+(4)[{\left(25\right)}^{2}]+(14n+22)[{\left(5\right)}^{2}]=(8n+20)[(36)]+(4)[(625)]+(14n+22)[(25)].=638n+3770.$$

• The third Revan index

$$\eta R_{3}(G)=\sum_{e=\mu \upsilon }[\tau (\mu )-\tau (\upsilon )]R_{3}(G)=(8n+20)[{\left(6-1\right)}^{2}]+(4)[{\left(5-5\right)}^{2}]+(14n+22)[{\left(5-1\right)}^{2}]=(8n+20)[{\left(5\right)}^{2}]+(4)[{\left(0\right)}^{2}]+(14n+22)[{\left(4\right)}^{2}]=(8n+20)[(25)]+(4)[(0)]+(14n+22)[(16)]=424n+852.$$

• The first Revan vertex index

$$R_{1}(G)=\sum_{e=\mu \upsilon }{\left[\tau (\bar{(}\mu )\right)}^{2}]R_{1}(G)=(8n+20)[{\left(6\right)}^{2}]+(14n+24)[{\left(5\right)}^{2}]+(6n+10)[{\left(1\right)}^{2}]=(8n+20)[(36)]+(14n+24)[(25)]+(6n+10)[(1)].=644n+1330.$$

A numerical comparison between different K-Banhatti indices is shown in Table 4.

**Table 4:** Degree-based Banhatti indices.

[*n*]	*B*_1_	*B*_2_	*HB*_1_	*HB*_2_	*H*_*b*_	*ηR*_1_	*ηR*_2_	*ηR*_3_	*R*_1_
[1]	1228	2740	12012	77868	33.4242	3068	4408	1974	1276
[2]	1644	3692	16180	105428	43.3787	3964	5046	2618	1700
[3]	2060	4644	20348	132988	53.3332	4860	5684	3262	2124
[4]	2476	5596	24516	160548	63.2877	5756	6322	3906	2548
[5]	2892	6548	28684	188108	73.2422	6652	6960	4550	2972
[6]	3308	7500	32852	215668	83.1967	7548	7598	5194	3396
[7]	3724	8452	37020	243228	93.1512	8444	8236	5838	3820
[8]	4140	9404	41188	270788	103.1057	9340	8874	6482	4244
[9]	4556	10356	45356	298348	113.0602	10236	9512	7126	4668
[10]	4972	11308	49524	325908	123.0147	11132	10150	7770	5092

**Figure 6:**
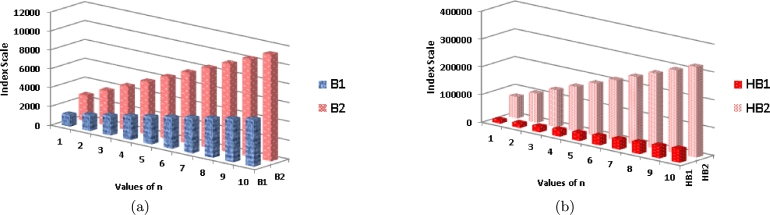
Graphical representation of (a) *B*_1_ and *B*_2_ (b) *HB*_1_ and *HB*_2_.

Analyzing Table 4 and Fig. 6(*a*), it is evident that as the value of *n* increases, the $B_{1}$ index demonstrates a significantly rapid growth compared to $B_{2}$. Similarly, in Fig. 6(*b*), it can be observed that with an increasing value of *n*, the $HB_{2}$ index exhibits a more pronounced growth rate in comparison to $HB_{1}$.

**Figure 7:**
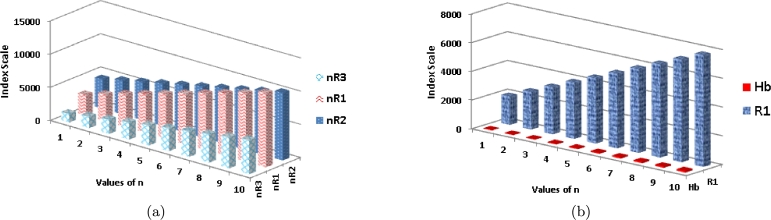
Graphical representation of (a) *ηR*_1_, *ηR*_2_ and *ηR*_3_ (b) *Hb* and *R*_1_.

Moving on to Table 4 and Fig. 7(*b*), it can be seen that as *n* increases, the $\eta R_{1}$ index shows a substantially higher growth rate compared to $H_{b}$ and $\eta R_{2}$. Likewise, in Fig. 7(*a*), it is apparent that as *n* increases, the $R_{1}$ index displays a more rapid growth rate compared to $R_{2}$ and $R_{3}$ indices.

## 5 Computing K-Banhatti entropy

In this section, we proceed to compute the entropy of the K-Banhatti index and assess the numerical results obtained. Furthermore, we present visual representations showcasing the response of each topological index to various parameter values. To achieve the desired outcomes, we employ a combination of strategies. By utilizing the computed K-Banhatti indices and corresponding values from Table 3 in Equation (1), we derive the following entropy values.

• The first K-Banhatti entropy

$$ENT_{B_{1}}=\log(B_{1})-\frac{1}{\left(B_{1}\right)}\log[\prod_{\mu e\in E(G)}{\left(d(\mu )+\theta_{\left(e\right)}\right)}^{\left(d(\mu )+\theta_{\left(e\right)}\right)}]ENT_{B_{1}}=\log(416n+812)-\frac{(8n+20)\log{\left(17\right)}^{17}}{\left(416n+812\right)}-\frac{(4)\log{\left(8\right)}^{8}}{\left(416n+812\right)}-\frac{(14n+22)\log{\left(20\right)}^{20}}{\left(416n+812\right)}$$

• The second K-Banhatti entropy

$$ENT_{B_{2}}=\log(B_{2})-\frac{1}{\left(B_{2}\right)}\log[\prod_{\mu e\in E(G)}{\left(d(\mu )\times \theta_{\left(e\right)}\right)}^{\left(d(\mu )\times \theta_{\left(e\right)}\right)}]ENT_{B_{2}}=\log(952n+1788)-\frac{(8n+20)\log{\left(35\right)}^{35}}{\left(952n+1788\right)}-\frac{(4)\log{\left(8\right)}^{8}}{\left(952n+1788\right)}-\frac{(14n+22)\log{\left(48\right)}^{48}}{\left(952n+1788\right)}$$

• The first hyper K-Banhatti entropy

$$ENT_{HB_{1}}=\log(HB_{1})-\frac{1}{\left(HB_{1}\right)}\log[\prod_{\mu e\in E(G)}{\left({\left(d(\mu )+\theta_{\left(e\right)}\right)}^{2}\right)}^{{\left(d(\mu )+\theta_{\left(e\right)}\right)}^{2}}]ENT_{HB_{1}}=\log(4168n+7844)-\frac{(8n+20)\log{\left(157\right)}^{157}}{\left(4168n+7844\right)}-\frac{(4)\log{\left(32\right)}^{32}}{\left(4168n+7844\right)}-\frac{(14n+22)\log{\left(208\right)}^{208}}{\left(4168n+7844\right)}$$

• The second hyper K-Banhatti entropy

$$ENT_{HB_{2}}=\log(HB_{2})-\frac{1}{\left(HB_{2}\right)}\log[\prod_{\mu e\in E(G)}{\left({\left(d(\mu )\times \theta_{\left(e\right)}\right)}^{2}\right)}^{{\left(d(\mu )\times \theta_{\left(e\right)}\right)}^{2}}]ENT_{HB_{2}}=\log(27560n+50308)-\frac{(8n+20)\log{\left(925\right)}^{925}}{\left(27560n+50308\right)}-\frac{(4)\log{\left(32\right)}^{32}}{\left(27560n+50308\right)}-\frac{(14n+22)\log{\left(1440\right)}^{1449}}{\left(27560n+50308\right)}$$

• The K-Banhatti harmonic entropy

$$ENT_{H_{b}}=\log(H_{b})-\frac{1}{\left(H_{b}\right)}\log[\prod_{\mu e\in E(G)}{\left(\frac{2}{d(\mu )+\theta_{\left(e\right)}}\right)}^{\left(\frac{2}{d(\mu )+\theta_{\left(e\right)}}\right)}]ENT_{H_{b}}=\log(9.9545n+23.4697)-\frac{(8n+20)\log{\left(\frac{17}{33}\right)}^{\frac{17}{33}}}{\left(9.9545n+23.4697\right)}-\frac{(4)\log{\left(\frac{4}{4}\right)}^{\frac{4}{4}}}{\left(9.9545n+23.4697\right)}-\frac{(14n+22)\log{\left(\frac{5}{12}\right)}^{\frac{5}{12}}}{\left(9.9545n+23.4697\right)}$$

• The first Revan entropy

$$ENT_{\eta R_{1}}={{\log(\eta R_{1})-\frac{1}{\left(\eta R_{1}\right)}\log[\prod_{\mu \upsilon \in E(G)}(\tau (\mu )+\tau (\upsilon ))}^{2})}^{{\left(\tau (\mu )+\tau (\upsilon )\right)}^{2}}]ENT_{\eta R_{1}}=\log(896n+2172)-\frac{(8n+20)\log{\left(49\right)}^{49}}{\left(896n+2172\right)}-\frac{(4)\log{\left(100\right)}^{100}}{\left(896n+2172\right)}-\frac{(14n+22)\log{\left(36\right)}^{36}}{\left(896n+2172\right)}$$

• The second Revan entropy

$$ENT_{\eta R_{2}}={{\log(\eta R_{2})-\frac{1}{\left(\eta R_{2}\right)}\log[\prod_{\mu \upsilon \in E(G)}(\tau (\mu )\times \tau (\upsilon ))}^{2})}^{{\left(\tau (\mu )\times \tau (\upsilon )\right)}^{2}}]ENT_{\eta R_{2}}=\log(638n+3770)-\frac{(8n+20)\log{\left(36\right)}^{36}}{\left(638n+3770\right)}-\frac{(4)\log{\left(625\right)}^{625}}{\left(638n+3770\right)}-\frac{(14n+22)\log{\left(25\right)}^{25}}{\left(638n+3770\right)}$$

• The third Revan entropy

$$ENT_{\eta R_{3}}=\log(\eta R_{3})-\frac{1}{\left(\eta R_{3}\right)}\log[\prod_{uv\in E(G)}{\left(\tau (\mu )-\tau (\upsilon )\right)}^{\left(\tau (\mu )-\tau (\upsilon )\right)}]ENT_{\eta R_{3}}=\log(424n+852)-\frac{(8n+20)\log{\left(25\right)}^{25}}{\left(424n+852\right)}-\frac{(4)\log{\left(0\right)}^{0}}{\left(424n+852\right)}-\frac{(14n+22)\log{\left(16\right)}^{16}}{\left(424n+852\right)}$$

• The first Revan vertex entropy

$$ENT_{R_{1}}={{\log(R_{1})-\frac{1}{\left(R_{1}\right)}\log[\prod_{\mu \upsilon \in E(G)}(\tau (\bar{(}\mu ))}^{2})}^{{\left(\tau (\bar{(}\mu )\right)}^{2})}]ENT_{R_{1}}=\log(644n+1330)-\frac{(8n+20)log{\left(36\right)}^{36}}{\left(644n+1330\right)}-\frac{(14n+24)\log{\left(25\right)}^{25}}{\left(644n+1330\right)}-\frac{(6n+10)\log{\left(1\right)}^{1}}{\left(644n+1330\right)}$$

**Figure 8:**
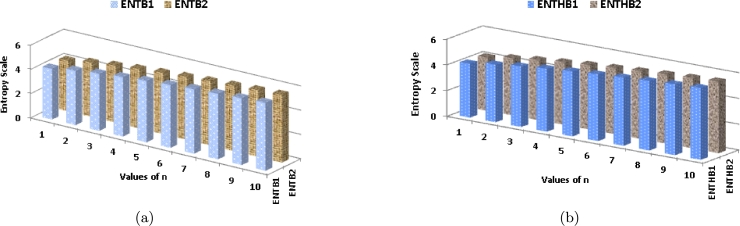
Graphical representation of (a) $ENT_{B_{1}}$ and $ENT_{B_{2}}$ (b) $ENT_{HB_{1}}$ and $ENT_{HB_{2}}$.

Analyzing Table 5 and Fig. 8(*a*), it becomes evident that as the value of *n* increases, the $EB_{1}$ index exhibits a significantly rapid growth compared to $EB_{2}$. Similarly, in Fig. 8(*b*), it can be observed that with an increasing value of *n*, the $EHB_{2}$ index demonstrates a more pronounced growth rate in comparison to $EHB_{1}$.

**Table 5:** Numerical comparison of entropy of K-Banhatti Indices.

[*n*]	$ENT_{B_{1}}$	$ENT_{B_{2}}$	$ENT_{HB_{1}}$	$ENT_{HB_{2}}$	$ENT_{H_{b}}$	$ENT_{\eta R_{1}}$	$ENT_{\eta R_{2}}$	$ENT_{\eta R_{3}}$	$ENT_{R_{1}}$
[1]	4.1821	4.1512	4.1790	4.1438	4.2560	4.2583	3.2633	4.2262	4.1563
[2]	4.4739	4.4495	4.4671	4.4378	4.5167	4.5145	3.6191	4.5086	4.4432
[3]	4.6994	4.6789	4.6904	4.6647	4.7232	4.7183	3.9091	4.7285	4.6659
[4]	4.8834	4.8653	4.8729	4.8495	4.8944	4.8875	4.1521	4.9087	4.8479
[5]	5.0387	5.0224	5.0272	5.0055	5.0405	5.0322	4.3596	5.0613	5.0018
[6]	5.1731	5.1582	5.1609	5.1404	5.1679	5.1586	4.5401	5.1937	5.1352
[7]	5.2915	5.2777	5.2788	5.2592	5.2809	5.2707	4.6991	5.3106	5.2529
[8]	5.3974	5.3844	5.3842	5.3654	5.3824	5.3716	4.8409	5.4152	5.3581
[9]	5.4932	5.4809	5.4796	5.4614	5.4746	5.4632	4.9684	5.5099	5.4533
[10]	5.5806	5.5688	5.5666	5.5491	5.5590	5.5471	5.0841	5.5964	5.5403

**Figure 9:**
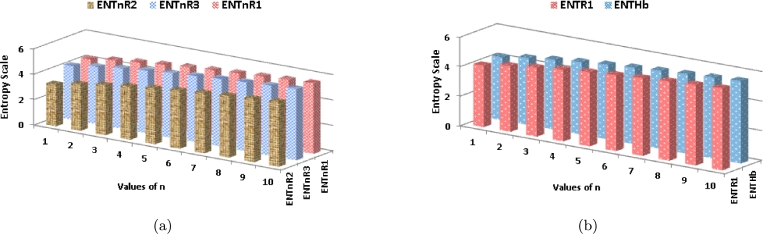
Graphical representation of (a) $ENT_{\eta R_{1}}$, $ENT_{\eta R_{2}}$ and $ENT_{\eta R_{3}}$ (b) *ENT*_*Hb*_ and *ENTR*_1_.

Moving on to Table 5 and Fig. 9(*a*), it is noticeable that as *n* increases, the $E\eta R_{1}$ index shows a substantially higher growth rate compared to $EH_{b}$ and $E\eta R_{2}$. Likewise, in Fig. 9(*b*), it is apparent that as *n* increases, the $ER_{3}$ index displays a more rapid growth rate in comparison to the $ER_{1}$ index.

## 6 Linear regression between K-Banhatti indices and Shannon entropy

**Figure 10:**
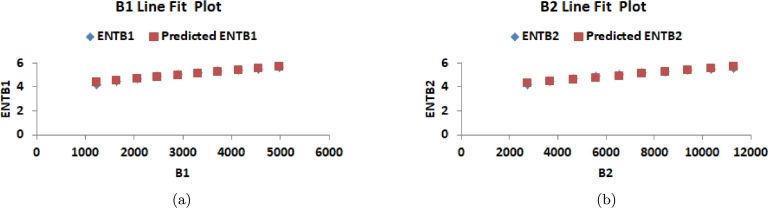
(a) Line fit plot between *B*_1_ and $ENT_{B_{1}}$ (b) Line fit plot between *B*_2_ and $ENT_{B_{2}}$.

**Figure 11:**
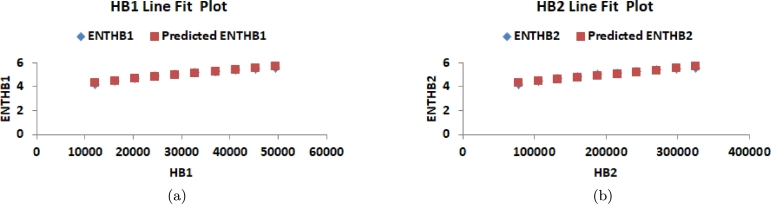
(a) Line fit plot between *HB*_1_ and $ENT_{HB_{1}}$ (b) Line fit plot between *HB*_2_ and $ENT_{HB_{2}}$.

**Figure 12:**
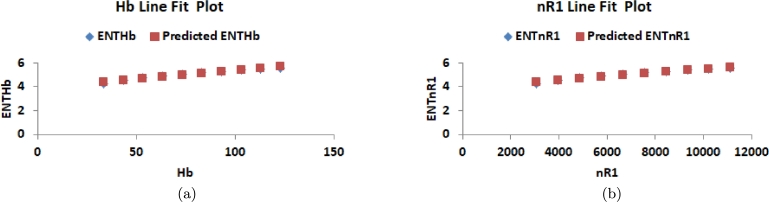
(a) Line fit plot between *Hb* and *ENT*_*Hb*_ (b) Line fit plot between *ηR*_1_ and $ENT_{\eta R_{1}}$.

**Figure 13:**
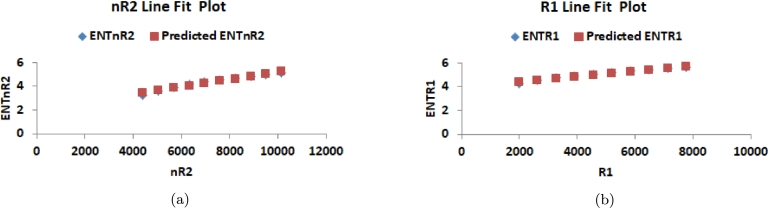
(a) Line fit plot between *ηR*_2_ and $ENT_{\eta R_{2}}$ (b) Line fit plot between *R*_1_ and $ENT_{R_{1}}$.

**Figure 14:**
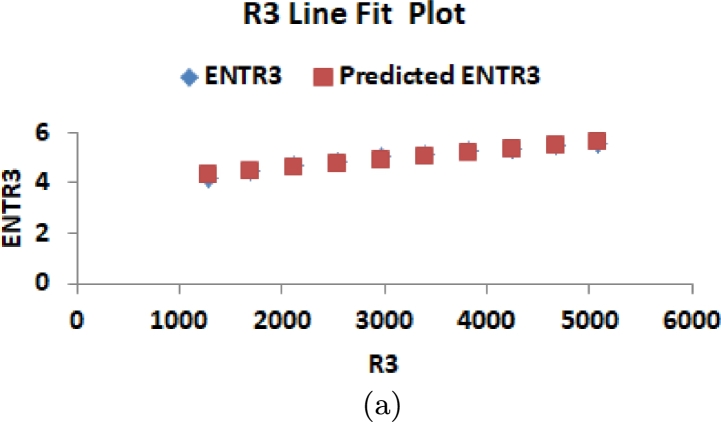
(a) Line fit plot between *ηR*_3_ and $ENT_{\eta R_{3}}$.

This section delves into the relationship between K-Banhatti indices and entropy values, examining their correlation. Researchers rely on graphical and numerical representations of these results to save time and minimize laboratory work. Consequently, we computed the degree-based entropies for various values of n in the $RhCl_{3}$ structures. The line fitting of entropy measure is shown in Fig. 10(a), Fig. 10(b), Fig. 11(a), Fig. 11(b), Fig. 12(a), Fig. 12(b), Fig. 13(a), Fig. 13(b) and Fig. 14. By employing curve-fitting techniques, it becomes possible to explore the connection between various types of variables.

In this study, we applied this approach to examine the relationship between entropy formation and multiple indices. Through the manipulation of underlying factors, we utilized linear curve fitting to determine the degree of alignment between entropy and the indices. The accuracy of the analysis was assessed using metrics such as linear regression, standard error (*SE*) estimation, correlation coefficient (*R*), coefficient of determination ($R^{2}$), and coefficients *a* and *b*. Our primary focus was placed on highlighting the significance of $R^{2}$. The simulations were conducted using Microsoft Excel. Table 6 presents the correlation coefficient values between entropy and each of the indices, providing a quantitative measure of their significance. Additionally, Table 7 showcases the predicted entropy values obtained from the analysis.

**Table 6:** Statistical parameter of linear model.

*ENT*_*I*_	*a*	*b*	*R*	*R*^2^	*SE*	*F*
$ENT_{B_{1}}$	0.000357953	3.911728585	0.981655102	0.963646739	0.091991818	207.6981223
$ENT_{B_{2}}$	0.00015838	3.891321292	0.980780824	0.961931025	0.096323713	202.1448726
$ENT_{HB_{1}}$	3.54761*E*05	3.919140064	0.981583114	0.963505409	0.092412953	211.2105703
$ENT_{HB_{2}}$	5.42709*E* − 06	3.892010057	0.981115135	0.962586908	0.094693438	205.828892
*ENT*_*HB*_	0.014012806	3.93352869	0.983683128	0.967632497	0.081926786	239.1614798
						
$ENT_{\eta R_{2}}$	0.000306961	2.109189807	0.983858319	0.967977191	0.114388725	241.8219307
$ENT_{\eta R_{3}}$	0.000226922	3.940404178	0.982006944	0.964337639	0.090247305	216.3261456
$ENT_{R_{1}}$	0.000347847	3.878007058	0.981655102	0.963646739	0.091991818	212.0627914

**Table 7:** Predicted Graph entropy.

[*n*]	$ENT_{B_{1}}$	$ENT_{B_{2}}$	$ENT_{HB_{1}}$	$ENT_{HB_{2}}$	$ENT_{H_{b}}$	$ENT_{\eta R_{1}}$	$ENT_{\eta R_{2}}$	$ENT_{R_{1}}$	$ENT_{\eta R_{3}}$
[1]	4.3512	4.3252	4.3452	4.3146	4.4018	4.4009	3.4622	4.3883	4.3218
[2]	4.5002	4.4760	4.4931	4.4641	4.5413	4.5390	3.6581	4.5344	4.4693
[3]	4.6491	4.6268	4.6410	4.6137	4.6808	4.6770	3.8539	4.6806	4.6168
[4]	4.7980	4.7776	4.7888	4.7633	4.8203	4.8151	4.0497	4.8267	4.7643
[5]	4.9469	4.9283	4.9367	4.9128	4.9598	4.9532	4.2456	4.9728	4.9118
[6]	5.0958	5.0791	5.0846	5.0624	5.0993	5.0913	4.4414	5.1190	5.0592
[7]	5.2447	5.2299	5.2324	5.2120	5.2388	5.2293	4.6373	5.2651	5.2067
[8]	5.3936	5.3807	5.3803	5.3616	5.3783	5.3674	4.8331	5.4113	5.3542
[9]	5.5425	5.5315	5.5281	5.5111	5.5178	5.5055	5.0290	5.5574	5.5017
[10]	5.6914	5.6822	5.6760	5.6607	5.6573	5.6435	5.2248	5.7035	5.6492

In Table 6 and Fig. 14, the index $\eta R_{3}$ exhibits the highest values for both *R* and $R^{2}$, indicating a strong correlation with entropy. Additionally, it has the lowest value for standard error (SE) in comparison to the other indices. Therefore, based on these criteria, $\eta R_{1}$ can be considered the best predictor of entropy.

Table 5 displays the entropy values obtained using Eq. (1), while Table 7 presents the predicted entropy values obtained through linear regression models.

## 7 Conclusion

In this article, we computed K-Banhatti indices. Using K-Banhatti topological descriptors of $RhCl_{3}$, we generalized analytical expressions. Then use it in entropy formulas and find the entropies of K-Banhatti indices of $RhCl_{3}$. Following a considerable increase in disorder, the transition state of $RhCl_{3}$ with rising *n* values exhibits the biggest modification in entropy.

By employing a line fit technique, we were able to explore the relationship between entropy formation and various indices, thereby enabling the investigation of connections among multiple types of variables. This analysis involved utilizing the line fit method to estimate the correlation between entropy and the K-Banhatti index while manipulating several underlying parameters. To assess the accuracy of our findings, we employed various evaluation metrics such as linear regression, standard error estimation, *R*, $R^{2}$, and coefficient analysis (specifically coefficients a and b). Emphasis was primarily placed on the significance indicated by the $R^{2}$ value. According to the results of the investigation into these structures; in this regard, the First Revan K-Banhatti index consistently delivers the most optimal and progressively enhancing outcomes due to its superior performance of $R^{2}$ and *F*. All the obtained results were presented both in numerical form and through graphical representations.

## CRediT authorship contribution statement

**Mazhar Hussain:** Conceptualization, Data curation, Formal analysis, Funding acquisition. **Muhammad Kamran Siddiqui:** Formal analysis, Investigation, Project administration, Software, Supervision, Writing – original draft. **Muhammad Farhan Hanif:** Formal analysis, Investigation, Methodology, Software, Validation, Visualization. **Hasan Mahmood:** Data curation, Formal analysis, Investigation, Software, Validation. **Zohaib Saddique:** Data curation, Formal analysis, Resources, Validation, Writing – review & editing. **Samuel Asefa Fufa:** Conceptualization, Data curation, Funding acquisition, Investigation, Supervision, Validation, Visualization, Writing – review & editing.

## Declaration of Competing Interest

All authors declare that there are no conflicts of interest regarding the publication of this paper.

## Data Availability

No data was used for the research described in the article.

## References

[1] Amma J.P., Stille J.K. (1982). Chiral 1, 2-diphosphine ligands. Synthesis and application to rhodium-catalyzed asymmetric hydrogenations. J. Org. Chem..

[2] Anjum M.S., Safdar M.U. (2019). K Banhatti and K hyper-Banhatti indices of nanotubes. Eng. Appl. Sci. Lett..

[3] Arockiaraj M., Kavitha S.R.J., Mushtaq S., Balasubramanian K. (2020). Relativistic topological molecular descriptors of metal trihalides. J. Mol. Struct..

[4] Arockiaraj M., Paul D., Ghani M.U., Tigga S., Chu Y.M. (2023). Entropy structural characterization of zeolites BCT and DFT with bond-wise scaled comparison. Sci. Rep..

[5] Arockiaraj M., Jency J., Mushtaq S., Shalini A.J., Balasubramanian K. (2023). Covalent organic frameworks: topological characterizations, spectral patterns and graph entropies. J. Math. Chem..

[6] Baig A.Q., Naeem M., Gao W. (2018). Revan and hyper-Revan indices of octahedral and icosahedral networks. Appl. Math. Nonlinear Sci..

[7] Beyer H., Emmerich J., Chatziapostolou K., Koehler K. (2011). Decomposition of nitrous oxide by rhodium catalysts: effect of rhodium particle size and metal oxide support. Appl. Catal. A, Gen..

[8] Brauer G. (2012).

[9] Chen Z., Dehmer M., Shi Y. (2014). A note on distance-based graph entropies. Entropy.

[10] Cotton S. (1997).

[11] Dehmer M. (2008). Information processing in complex networks: graph entropy and information functionals. Appl. Math. Comput..

[12] Dehmer M., Mowshowitz A. (2011). A history of graph entropy measures. Inf. Sci..

[13] Giordano G., Crabtree R.H., Heintz R.M., Forster D., Morris D.E. (1979). Di-chloro-bis (1, 5-cyclooctadlene) dirhodium (I). Inorg. Synth..

[14] Greenwood N.N., Earnshaw A. (2012).

[15] Ibers J.A., Snyder R.G. (1962). Structure of (rhodium (I) chloride-1, 5-cycloöctadiene) 2. J. Am. Chem. Soc..

[16] Jayakumar J., Parthasarathy K., Cheng C.H. (2012). One-pot synthesis of isoquinolinium salts by rhodium-catalyzed bond activation: application to the total synthesis of oxychelerythrine. Angew. Chem..

[17] Kavitha S.R.J., Abraham J., Arockiaraj M., Jency J., Balasubramanian K. (2021). Topological characterization and graph entropies of tessellations of kekulene structures: existence of isentropic structures and applications to thermochemistry, nuclear magnetic resonance, and electron spin resonance. J. Phys. Chem. A.

[18] Kulli V.R. (2016). First multiplicative K Banhatti index and coindex of graphs. Ann. Pure Appl. Math..

[19] Kulli V.R. (2016). On multiplicative K Banhatti and multiplicative K hyper-Banhatti indices of V-Phenylenic nanotubes and nanotorus. Ann. Pure Appl. Math..

[20] Kulli V.R. (2016). On K Banhatti indices of graphs. J. Comput. Math. Sci..

[21] Kulli V.R., On K. (2016). On K hyper-Banhatti indices and coindices of graphs. Int. Res. J. Pure Algebra.

[22] Kulli V.R. (2020). Harmonic Zagreb-K-Banhatti index of a graph. Int. J. Math. Trends Technol..

[23] Kulli V.R. (2017). Revan indices of oxide and honeycomb networks. Int. J. Math. Appl..

[24] Lau K.S.Y., Becker Y., Huang F., Baenziger N., Stille J.K. (1977). Mechanism of decarbonylation of acid chlorides with chlorotris (triphenylphosphine) rhodium (I) structure and stereochemistry. J. Am. Chem. Soc..

[25] Lautens M., Fagnou K., Yang D. (2003). Rhodium-catalyzed asymmetric ring opening reactions of oxabicyclic alkenes: application of halide effects in the development of a general process. J. Am. Chem. Soc..

[26] Manzoor S., Siddiqui M.K., Ahmad S., Fufa S.A. (2022). On computation of entropy measures and molecular descriptors for isomeric natural polymers. J. Math..

[27] McCleverty J.A., Wilkinson G., Lipson L.G., Maddox M.L., Kaesz H.D. (1966). Dichlorotetracarbonyldirhodium. Inorg. Synth..

[28] Morowitz H.J. (1955). Some order-disorder considerations in living systems. Bull. Math. Biophys..

[29] Mowshowitz A., Dehmer M. (2012). Entropy and the complexity of graphs revisited. Entropy.

[30] Osborn J.A., Jardine F.H., Young J.F., Wilkinson G. (1966). The preparation and properties of tris (triphenylphosphine) halogenorhodium (I) and some reactions thereof including catalytic homogeneous hydrogenation of olefins and acetylenes and their derivatives. J. Chem. Soc., A: Inorg., Phys., Theor..

[31] Rehman A., Nawaz M. (2018). K Banhatti and K hyper Banhatti indices of the line graphs of H-Pantacenic nanotubes. J. Prime Res. Math..

[32] Renner H., Schlamp G., Kleinwächter I., Drost E., Lüschow H.M., Tews P. (2000). Platinum group metals and compounds. Ullmann's Encycl. Ind. Chem..

[33] Roodt A., Otto S., Steyl G. (2003). Structure and solution behaviour of rhodium (I) Vaska-type complexes for correlation of steric and electronic properties of tertiary phosphine ligands. Coord. Chem. Rev..

[34] Siddiqui M.K., Imran M., Ahmad A. (2016). On Zagreb indices, Zagreb polynomials of some nanostar dendrimers. Appl. Math. Comput..

[35] Van't Blik H.F.J., Van Zon J.B.A.D., Huizinga T., Vis J.C., Koningsberger D.C., Prins R. (1985). Structure of rhodium in an ultradispersed rhodium/alumina catalyst as studied by EXAFS and other techniques. J. Am. Chem. Soc..

[36] White C., Yates A., Maitlis P.M., Heinekey D.M. (1992). (Pentamethylcyclopentadienyl) rhodium and-iridium compounds. Inorg. Synth..

[37] Zhao D., Siddiqui M.K., Javed S., Sherin L., Kausar F. (2020). Molecular topological indices-based analysis of thermodynamic properties of graphitic carbon nitride. Eur. Phys. J. Plus.

[38] Wang W., Naeem M., Rauf A., Riasat A., Aslam A., Anoh Yannick K. (2021). On analysis of Banhatti indices for hyaluronic acid curcumin and hydroxychloroquine. J. Chem..

[39] Zhang Xiujun, Muhammad Awais Hafiz, Javaid Muhammad, Kamran Siddiqui Muhammad (2019). Multiplicative Zagreb indices of molecular graphs. J. Chem..

[40] Zhang X., Jiang H., Liu J.B., Shao Z. (2018). The Cartesian product and join graphs on edge-version atom-bond connectivity and geometric arithmetic indices. Molecules.

[41] Zhang X., Rauf A., Ishtiaq M., Siddiqui M.K., Muhammad M.H. (2022). On degree based topological properties of two carbon nanotubes. Polycycl. Aromat. Compd..

[42] Zhang X., Naeem M., Baig A.Q., Zahid M.A. (2021). Study of hardness of superhard crystals by topological indices. J. Chem..

[43] Zhang X., Siddiqui M.K., Javed S., Sherin L., Kausar F., Muhammad M.H. (2022). Physical analysis of heat for formation and entropy of Ceria Oxide using topological indices. Comb. Chem. High Throughput Screen..

[44] X. Zhang, H.G. Reddy, A. Usha, M.C. Shanmukha, M. Reza Farahani, M. Alaeiyan, A study on anti-malaria drugs using degree-based topological indices through QSPR analysis, 2022. 10.3934/mbe.2023167 36899594

